# Cyclooxygenase inhibition lowers prostaglandin E_2 _release from articular cartilage and reduces apoptosis but not proteoglycan degradation following an impact load *in vitro*

**DOI:** 10.1186/ar2346

**Published:** 2007-12-20

**Authors:** Janet E Jeffrey, Richard M Aspden

**Affiliations:** 1 Department of Orthopaedic Surgery, University of Aberdeen, Institute of Medical Sciences, Foresterhill, Aberdeen, AB25 2ZD, UK

## Abstract

This study investigated the release of prostaglandin E_2 _(PGE_2_) from cartilage following an impact load *in vitro *and the possible chondroprotective effect of cyclooxygenase-2 (COX-2) inhibition using non-steroidal anti-inflammatory drugs (NSAIDs).

Explants of human articular cartilage were subjected to a single impact load in a drop tower, and then cultured for 6 days in the presence of either a selective COX-2 inhibitor (celecoxib; 0.01, 0.1, 1.0 and 10 μM) or a non-selective COX inhibitor (indomethacin; 0.1 and 10 μM). The concentrations of PGE_2 _and glycosaminoglycans (GAGs), a measure of cartilage breakdown, were measured in the explant culture medium at 3 and 6 days post-impact. Apoptotic cell death was measured in frozen explant sections by the terminal deoxynucleotidyl transferase-mediated dUTP nick-end labelling (TUNEL) method.

PGE_2 _levels were increased by more than 20-fold in the medium of explants at both 3 (p = 0.012) and 6 days (p = 0.004) following impact, compared with unloaded controls. In the presence of celecoxib and indomethacin, the PGE_2 _levels were reduced in a dose-related manner. These inhibitors, however, had no effect in reducing the impact-induced release of GAGs from the cartilage matrix. Addition of celecoxib and indomethacin significantly reduced the number of trauma-induced apoptotic chondrocytes in cartilage explant sections.

In this study, a marked increase in PGE_2 _was measured in the medium following an impact load on articular cartilage, which was abolished by the selective COX-2 inhibitor, celecoxib, and non-selective indomethacin. These inhibitors reduced chondrocyte apoptosis but no change was observed in the release of GAGs from the explants, suggesting that the COX/PGE_2 _pathway is not directly responsible for cartilage breakdown following traumatic injury. Our *in vitro *study demonstrates that it is unlikely that COX-2 inhibition alone would slow down or prevent the development of secondary osteoarthritis.

## Introduction

Articular cartilage is a highly specialised connective tissue that covers the ends of long bones in diarthrodial joints. The tissue protects the joint by distributing applied loads and providing a low-friction, wear-resistant, lubricated surface to facilitate movement. The cartilage matrix consists of collagen fibres that reinforce a proteoglycan gel. The main protoeoglycan is aggrecan, which comprises a protein core highly substituted with polysulfated glycosaminoglycan (GAG) side chains.

Traumatic joint damage, such as may be sustained in a road traffic accident or a sporting injury, is a known risk factor for the subsequent development of secondary osteoarthritis (OA) [1]. Injury can result in progressive cartilage loss causing pain, swelling, inflammation and joint immobility. Ultimately, a joint replacement may be required. However the processes resulting in cartilage breakdown following injury and the ability of the tissue to repair itself are poorly understood. In humans, studies have shown elevated levels of breakdown products from cartilage matrix many years after injury [2-4]. The relationship between joint injury and OA development has also been demonstrated in various animal models both *in vivo *and *in vitro *[5,6].

Under normal physiological loading, articular cartilage is subjected to a variety of stresses. These biomechanical factors are believed to stimulate chondrocyte metabolism, providing a mechanism for the cartilage to adapt to the demands imposed by the body. However, in abnormal or injurious joint loading the balance between cartilage matrix synthesis and degradation is disturbed [7], resulting in tissue breakdown and the risk of progression of OA.

There is considerable evidence that the cytokine interleukin-1 (IL-1) plays an important role in OA, being up-regulated in OA synovium and cartilage [8,9]. IL-1β expression in articular cartilage is also regulated by mechanical factors [10]. It induces a catabolic cascade involving the cyclooxygenase (COX) enzymes; two isoforms of which, COX-1 and COX-2, catalyse the conversion of arachidonic acid to prostaglandins (PG), the major pro-inflammatory product being prostaglandin E_2 _(PGE_2_) [11]. COX-1 is the constitutive form of the enzyme, naturally expressed at low levels and essential to the normal function of many tissues, whereas COX-2 is the inducible form, which is commonly up-regulated following an insult to the tissue [12]. Consequently, PGE_2 _has been found to be elevated in cartilage, synovium and synovial fluid in OA joints [13,14] and also in normal cartilage by prolonged static mechanical loads [15]. Similarly COX-2, but not COX-1, has been shown to be up-regulated in chondrocytes of OA cartilage [16]. This COX-2/PGE_2 _pathway is of major interest in OA as the first line of treatment in this disease is the use of the non-steroidal anti-inflammatory drugs (NSAIDs) for pain relief. These drugs inhibit the activity of COX [17]. The non-selective NSAIDs inhibit both COX-1 and COX-2 (e.g. indomethacin) and more recently NSAIDs have been developed that are more selective for COX-2 (e.g. celecoxib) exhibiting fewer unwanted side effects.

Several studies, both in animal models [18] and in human joints [19,20], have shown that apoptosis (programmed cell death) is an important factor in the progression of OA. A positive correlation exists between severity of OA and percentage of apoptotic cells [21]. Apoptosis occurs following mechanical injury [22-26] and is thought to be initiated by IL-1β (in the presence of tumour necrosis factor (TNF) α; [27]), which in turn activates the caspase cascade [28]. As IL-1β is also involved in activating the COX/PGE_2 _pathway and PGE_2 _is reported to induce apoptosis in bovine articular chondrocytes [29], the prostanoid may have a role in the increased apoptosis observed following trauma.

In an attempt to understand the physical and biochemical changes occurring in articular cartilage following trauma, an *in vitro *impact model has been developed [7,30]. This consists of an instrumented drop tower, which enables an explant of cartilage to be subjected to a controlled impact load [31].

The aim of this study was to ascertain whether PGE_2 _was released by articular cartilage chondrocytes following an impact load, and whether COX-2 inhibition using NSAIDs could provide a chondroprotective role by preventing matrix degradation. In addition, the role of COX inhibitors on trauma-induced chondrocyte apoptosis was investigated.

## Materials and methods

### Cartilage explants

Human articular cartilage was obtained from femoral heads retrieved during hemiarthroplasty for fractured neck of femur within 12 h of surgery. Local Ethics Committee approval was granted for this procedure. Full depth, circular explants (5 mm diameter) of articular cartilage were removed from the underlying subchondral bone of human femoral heads (*n *= 3; ages 58, 63 and 68 years) using a cork borer and scalpel, and cultured in Dulbecco's modified Eagle's medium (DMEM; Gibco, Paisley, UK) supplemented with 10% foetal calf serum (Globepharm, Guildford, UK), 100 IU/ml penicillin, 100 μg/ml streptomycin, 0.25 μg/ml amphotericin B (Gibco) and 25 μg/ml ascorbic acid (Sigma-Aldrich Co. Ltd., Gillingham, UK). To minimise the effect of site variation and differing cartilage thicknesses, test and control explants were taken from adjacent sites over the femoral head. In this study, cartilage was removed from the underlying bone as the subchondral bone is often too fragile and the surface too uneven for impact loading. Following removal, and prior to impact loading, each explant was placed in 2 ml of culture medium in a 24-well plate and placed in an incubator at 37°C with 5% CO_2_. The explants were allowed to equilibrate for 72 h as Fermor *et al*. showed that there was an initial increase in PGE_2 _release from harvested cartilage explants, and that this stabilised after 72 h in culture and remained stable for up to 7 days [32]. The wet weight of each explant was measured prior to loading by placing in a sterile pre-weighed microcentrifuge tube containing DMEM.

### Impact loading

A specially designed drop tower was used to drop a mass on to a cartilage explant from a known height [7,30,31]. The explants were placed individually on the loading platen and subjected to a single impact load using a 500 g mass dropped from a height of 25 mm. The duration of each impact was approximately 3 ms, with an energy of 0.13 J and a peak stress of around 25 MPa. Impact loading conditions were chosen to produce moderate, but not overly severe, damage to the tissue based on our previous studies [7,30,33]. Control explants were placed in the machine but not loaded. Following impact, both control and loaded explants were re-cultured in fresh medium (1 ml per explant) for 6 days in the presence of either the selective COX-2 inhibitor celecoxib (donated by Pfizer Inc., New York, USA; 0.01, 0.1, 1.0 and 10 μM) or indomethacin (Sigma; 0.1 and 10 μM), which is non-selective and inhibits both COX-1 and COX-2. The experiment was performed three times, this being enough to obtain sufficient statistical power; each femoral head yielded eight treatment groups, each containing at least five explants (i.e. unloaded controls, loaded without inhibitor, four loaded with different concentrations of celecoxib and two loaded with different concentrations of indomethacin added to the medium). Ethanol was used as the solvent for the NSAIDs with a final concentration in the medium of <0.01% v/v. Ethanol was added at the same concentration to the medium of the control explants. The ranges of inhibitor concentrations in the culture medium were chosen to include peak plasma levels of drug found *in vivo*. This was 1.8 μM for celecoxib following a single dose of 200 mg and 5.6–8.4 μM for indomethacin [34]. After 3 days the culture medium was collected. Fresh medium, containing inhibitors as before, was added to each culture well for a further 3 days. The medium samples collected at days 3 and 6 were stored at -20°C.

### PGE_2 _release assay

PGE_2 _production was measured in the explant culture medium using a commercially available enzyme-linked immunosorbent assay (ELISA) kit (Prostaglandin E_2 _immunoassay, R&D Systems Ltd., Abingdon, UK). Results shown are measurements of total PGE_2 _synthesis after 3 days and after 6 days (combining the two 3-day periods).

### Glycosaminoglycan release assay

The concentration of GAGs, a measure of cartilage breakdown, was determined in the culture medium using the 1,9-dimethylmethylene blue (DMMB) assay [35]. The method used was modified from that described by Stone *et al*. [36] for use in a 96-well plate. Standard curves were obtained using concentrations of chondroitin 6-sulfate (Sigma) from 0–150 μg/ml at 10 μg/ml intervals. Duplicate aliquots (10 μl) of explant culture medium and standards were mixed with 200 μl of DMMB working solution in a 96-well plate, and the absorbance read at 525 nm using a Dynatech MR5000 spectrophotometer (Dynex Technologies Ltd., Worthing, UK) plate reader 3 min after the addition of the dye. Biolinx software was used to generate a standard curve and determine the concentration of GAG in each medium sample. PGE_2 _and GAG concentrations were normalised to the wet weight of each explant and expressed per mg of cartilage.

### Apoptosis detection

Articular cartilage explants were removed after 6 days in culture following impact load. They were snap frozen in OCT embedding medium (Raymond A Lamb Ltd., Eastbourne, UK) in isopentane cooled over liquid nitrogen. Four frozen (8 μm) cartilage sections were cut from each explant, collected and air dried onto SuperFrost Plus microscope slides (VWR International, Lutterworth, UK). Apoptosis was evaluated by TUNEL (terminal deoxynucleotidyl transferase-mediated dUTP nick-end labelling) staining using an ApoAlert DNA fragmentation assay kit (BD Biosciences Clontech, Palo Alto, CA, USA) following the manufacturer's protocol. Following TUNEL staining, the sections were stained with propidium iodide (PI, 20 μg/ml; Sigma) for 8 min at room temperature to counterstain the nuclei red. Positive control sections were treated with DNase I (1 μg/ml; Roche Diagnostics Ltd., Lewes, UK) for 10 min before TUNEL staining and negative control sections were treated with the nucleotide mix minus the TdT enzyme. Following TUNEL staining, all sections were washed and covered with Vectorshield fluorescent mounting medium (Vector Laboratories Inc., Peterborough, UK). Images of each section were taken, using the appropriate filters, with a digital camera (CoolSNAP, Roper Scientific GmbH, Germany.) attached to a fluorescence microscope (Zeiss, Welwyn Garden City, UK). The percentage of apoptotic cells was determined in each of the four sections for each explant by first counting the green (fluorescein) apoptotic cells followed by the total cell count (PI, red) with the aid of image analysis software (Image J, NIH, Bethesda, MD, USA). In order to validate the TUNEL method, some sections were stained with haematoxylin and eosin and the percentage of apoptotic cells counted by observing the morphology of the nucleus and cell membrane.

### Statistical analysis

**Figure 1:**
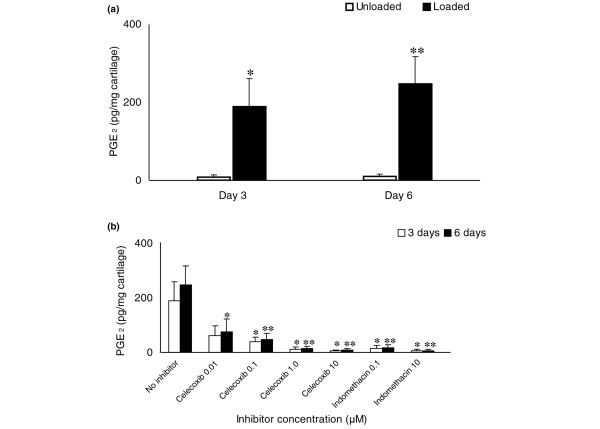
Release of PGE_2 _into culture medium following an impact load on articular cartilage explants. Explants of articular cartilage were impact loaded with a mass of 500 g dropped from 25 mm (0.13 J). The culture medium from each explant was collected at 3 days and 6 days following loading. The concentration of PGE_2 _in the medium was measured by ELISA, normalised to the wet weight of each explant and expressed per mg of cartilage. Day 6 results represent the cumulative release of PGE_2 _(combining the two 3-day periods). Mean values (± standard deviation, *n *= 3) from three experiments are shown. Each experiment used five replicates in each group. **(a) **Impact load significantly increased the concentration of PGE_2 _in the explant culture medium. Asterisks denote significant differences (*p ≤ 0.05, **p ≤ 0.01) between unloaded controls and impact loaded explants. **(b) **Celecoxib and indomethacin both reduced the release of PGE_2 _following an impact load. Asterisks denote significant differences (*p ≤ 0.05, **p ≤ 0.01) between impact loaded explants with no inhibitor added and impact loaded explants with inhibitor.

**Figure 2:**
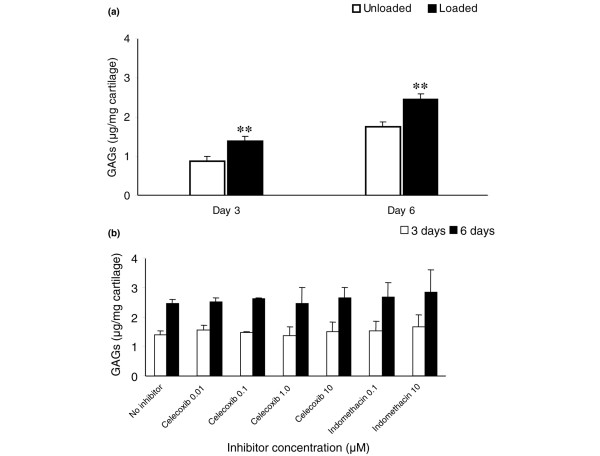
Release of glycosaminoglycans into the culture medium following an impact load on articular cartilage explants. Explants of human articular cartilage were impact loaded with a mass of 500 g dropped from 25 mm (0.13 J). The culture medium from each explant was collected at 3 days and 6 days following loading. The concentration of GAGs in the medium was measured with the DMMB assay, normalised to the wet weight of each explant and expressed per mg of cartilage. Day 6 results represent the cumulative release of GAGs (combining the two 3-day periods). Mean values (± standard deviation, *n *= 3) from three experiments are shown. Each experiment used five replicates in each group. **(a) **Impact load significantly increased the concentration of GAGs in the explant culture medium. Asterisks denote significant differences (**p ≤ 0.01) between unloaded controls and impact loaded explants. **(b) **The release of GAGs was unaffected by the addition of celecoxib or indomethacin.

**Figure 3:**
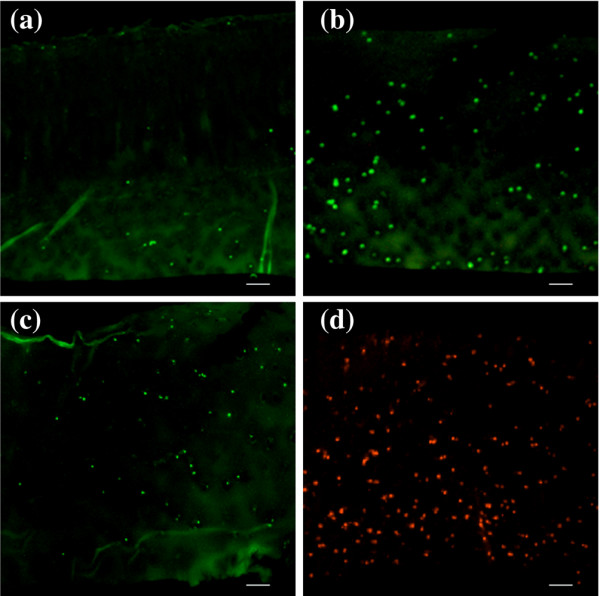
Determination of percentage of apoptotic cells in sections of articular cartilage using the TUNEL method. Cartilage explants were cultured for 2 days, impact loaded and then cultured for a further 6 days before being frozen, sectioned (8 μm) and TUNEL stained. **(a) **Unloaded control showed very few TUNEL positive cells. **(b) **The percentage of apoptotic cells increased significantly following an impact load. **(c) **Addition of celecoxib (0.1 μM) to the culture medium decreased the number of TUNEL positive cells in impact loaded explants. **(d) **The total cell count for each section was determined by counterstaining the nuclei red with propidium iodide. The bar represents 100 μm in all panels.

**Figure 4:**
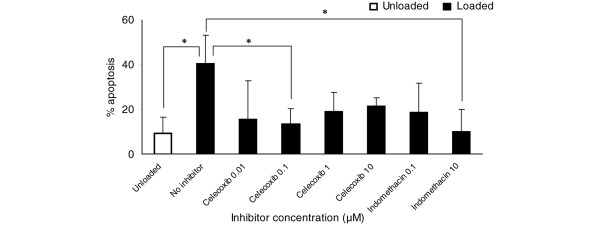
Celecoxib and indomethacin reduced the percentage of impact-induced apoptotic chondrocytes following an impact load. Following loading (as described in Figure 3) and culture in the presence of celecoxib and indomethacin for 6 days, the percentage of apoptotic cells in frozen sections (8 μm) of articular cartilage explants was measured using the TUNEL assay. Mean values (± standard deviation, *n *= 12). Asterisks denote significant differences between the groups as shown. (*p ≤ 0.05).

Differences in PGE_2_, GAG release and percentage apoptosis between treatment groups were assessed using the unpaired Student t test (two-tailed) using SPSS v.14 software (SPSS Inc., Chicago, IL, USA). A p value less than 0.05 was considered significant. In Figures 1, 2, 3, 4, asterisks denote significant differences; *p ≤ 0.05, **p ≤ 0.01. All data are expressed as mean ± standard deviation (SD).

## Results

PGE_2 _levels were increased 22-fold in the medium of explants at 3 days (p = 0.012) following impact compared to unloaded controls. By 6 days this increased further to 27-fold (p = 0.004) (Figure 1a). In the presence of celecoxib and indomethacin, the PGE_2 _levels were reduced in a dose-related manner both at 3 days and, more significantly, 6 days (Figure 1b). At the highest concentration (10 μM) the levels were reduced to those of the unloaded controls by both inhibitors at both timepoints. The baseline concentrations in the medium of control human cartilage explants were similar to those in other *in vitro *studies [37-39].

The concentration of GAGs in the medium was significantly higher in the loaded explants than in the unloaded controls at both day 3 (p = 0.010) and day 6 (p = 0.003) following impact (Figure 2a). However the addition of celecoxib or indomethacin to the culture medium had no effect on the release of GAGs from the cartilage matrix at either 3 days or 6 days (Figure 2b). Therefore, in this study, COX inhibition following impact load did not prevent proteoglycan depletion of cartilage.

An impact load increased the percentage of apoptotic cells compared with unloaded controls (p = 0.022). The apoptotic chondrocytes were evenly distributed throughout the zones of the loaded cartilage sections (Figure 3). Addition of the COX inhibitors to the medium reduced the percentage of impact-induced apoptosis at all doses and reached significance at 0.1 μM celecoxib (p = 0.034) and 10 μM indomethacin (p = 0.032) (Figure 4).

## Discussion

A single impact load resulted in a marked increase in PGE_2 _release, the number of apoptotic cells, and the concentration of GAGs in the culture medium. Could the elevated levels of PGE_2 _lead directly to these other changes and hence provide a target for early therapeutic intervention to delay or, even, prevent later secondary OA?

Tissue damage resulted in an increase in PGE_2 _released to the culture medium so that by day 3 after impact, the concentration was more than 20 times higher than in control groups. The detailed course over time of this was not studied, but release continued – albeit at a slower rate – over the next 3-day period. This release could be inhibited in a dose-dependent fashion by both celecoxib and indomethacin. Apoptosis was also reduced but not in the same pattern. Indomethacin halved the number of apoptotic cells at a concentration of 1 μM and halved it again at 10 μM. In contrast, celecoxib approximately halved the number of apoptoptic cells but concentration appeared to have little effect; maximal effect was found at 0.1 μM, but this was not significantly different from any of the other concentrations used. Despite the reduction in apoptosis, neither inhibitor had any effect on matrix degradation, as indicted by there being no change in GAG release.

The release of PGE_2 _by articular chondrocytes and its inhibition by indomethacin is well established [40]. Once released, however, the effects of this prostanoid on the cartilage matrix are rather unclear. PGE_2 _is reported to have anabolic effects on cartilage: increasing proteoglycan and collagen synthesis [41], stimulating proliferation and aggrecan synthesis [42], up-regulating glucocorticoid receptors [43] and, at low concentrations, stimulating collagen II synthesis. All these effects are possibly mediated by insulin-like growth factor 1 (IGF-1) via an autocrine loop [44]. However, this response was reported to be biphasic in chondrocytes *in vitro *because at higher concentrations of PGE_2 _collagen synthesis was reduced precipitously [44]. In earlier studies we showed that a static load of 1 MPa on human articular cartilage explants *in vitro *resulted in an increased expression of IL-1β [45] and PGE_2 _[15], though cyclic loading produced no measurable change in either IL-1β or PGE_2 _suggesting that this is a pathological response. In this study, we show that physical injury to cartilage following impact results in a significant increase in PGE_2_.

The increase in the percentage of apoptotic cells was similar to that we measured previously with human cartilage using the same drop-tower model [46]. The role of PGE_2 _in promoting apoptosis, however, remains unclear. Addition of exogenous PGE_2 _to bovine articular chondrocytes *in vitro *has been shown to cause apoptosis through a cAMP-dependent pathway [29]. However in human chondrocytes from OA cartilage, Notoya *et al*. [47] found that PGE_2 _had no effect on chondrocyte apoptosis itself, but the prostanoid enhanced apoptosis induced by exogenous nitric oxide and this effect could be prevented by COX-2 inhibition. In contrast, PGE_2 _has been reported to protect chondrocytes from apoptosis induced by actinomycin-D [48]. In addition, PGE_2 _is a chondrocyte growth inhibitor that requires NO for its production [49], and both PGE_2 _and NO are downstream mediators of IL-1. The partial, dose-independent reduction we found suggests that a cofactor role for PGE_2 _is possibly more likely than a direct effect. Further studies are required to investigate the role of NO and IL-1β.

Hashimoto *et al*. [21] linked chondrocyte apoptosis to matrix destruction. In this study however, the inhibition of apoptosis by the addition of celecoxib or indomethacin was not found to reduce the amount of GAGs, the breakdown products of cartilage proteoglycans, lost from loaded explants. This result is similar to that we previously found, culturing human articular cartilage explants with a broad spectrum caspase inhibitor (Z-VAD-FMK) reduced the percentage of impact-induced apoptotic chondrocytes but was unable to reduce the amount of GAGs released into the medium following impact [50]. Since articular cartilage is not vascularised and does not contain mononuclear phagocytes, there is no apparent mechanism for removal of apoptotic bodies following chondrocyte apoptosis. It has been shown that these apoptotic bodies express properties that contribute to pathologic matrix destruction [27]. Therefore, although matrix degradation, as measured by GAG release, was unchanged in this study, COX inhibition may still have chondroprotective effects by reducing the percentage of impact-induced apoptotic cells remaining in the tissue (and, therefore, there would be fewer potentially destructive apoptotic bodies). Together, though, these studies indicate that apoptosis alone is not driving matrix breakdown and that, once started, degradative enzymic activity may not be under direct cellular control. This may be because these enzymes are sequestered extracellularly in inactive forms and activation leads to a positive feedback pathway that is then out of direct control of the cells. Alternatively, enzymic activation is controlled by a different signalling pathway, though this then raises the question as to why the remaining cells cannot recognise this activity and inhibit it? The cells have been shown to be able to increase their levels of matrix biosynthesis following impact-induced damage [7] so perhaps matrix breakdown is part of a repair response to try to remove damaged tissue and replace it. Assuming, however, that it is important clinically to reduce matrix degradation, a two-pronged approach to treating damaged tissue would then be required; one agent to rescue the cells from apoptosis and another to inhibit enzymic degradation. The complexity of these mechanisms requires further investigation, in particular addition of exogenous PGE_2 _and subsequent measurement of proteoglycan synthesis in this system would demonstrate any anabolic effects. However, we have shown that inhibiting PGE_2 _in impact-damaged cartilage at least partially prevented the increase in apoptotic cells otherwise found after 6 days.

Both celecoxib and indomethacin could abolish the increase in PGE_2_. Celecoxib is 375 times more selective for COX-2 than COX-1 [51] whereas indomethacin is generally considered to be non-selective. Several studies have shown that COX inhibitors have an effect on cartilage metabolism. COX-2 inhibition has no direct effect on normal, healthy cartilage, but in the presence of IL-1β or TNFα it restores proteoglycan turnover [52]. Additionally, Hajjaji *et al*. [53] found that celecoxib had a favourable effect on the metabolism of proteoglycans and hyaluronic acid in samples of OA cartilage *in vitro*. Non-selective NSAIDs have differing effects on cartilage metabolism. Some stimulate, some have no effect, and others – including indomethacin – inhibit matrix synthesis [54,55]. Mastbergen *et al*. [38] have shown that in OA cartilage, NSAIDs with low COX-2/COX-1 selectivity exhibit adverse effects whereas high COX-2/COX-1 selective NSAIDs either had no effect or had reparative properties. Since in our study the non-selective NSAID indomethacin inhibited impact-induced apoptosis, future experiments with an experimental selective COX-1 inhibitor (i.e. SC-560) may be of use to investigate the precise role of COX-1 in this impact model. In patients, selective COX-2 inhibition would appear to confer beneficial effects on articular cartilage metabolism while avoiding the harmful effects of COX-1 inhibition, such as gastric irritation and inhibition of matrix synthesis, though possible cardiovascular effects of COX-2 inhibition have yet to be resolved.

## Conclusion

This study has shown that an impact load on articular cartilage results in a marked increase in PGE_2 _synthesis. This increase could be abolished by both the selective COX-2 inhibitor, celecoxib, and by non-selective indomethacin. Chondrocyte apoptosis, induced by impact, was also reduced by COX-2 inhibition. No change was observed in the release of GAGs from the explants in the presence of these inhibitors, however, suggesting that the COX/PGE_2 _pathway is not directly responsible for cartilage breakdown following traumatic injury. The inhibition by COX inhibitors of PGE_2 _release following trauma may provide an opportunity for early clinical intervention to reduce cell death from apoptosis. Our *in vitro *study suggests that it is unlikely, however, that COX-2 inhibition alone would slow down or prevent the development of secondary osteoarthritis.

## Abbreviations

COX = cyclooxygenase; ELISA = enzyme-linked immunosorbent assay; GAG = glycosaminoglycan; IL = interleukin; MMP = matrix metalloproteinase; iNOS = inducible nitric oxide synthase; NSAID = non-steroidal anti-inflammatory drug; OA = osteoarthritis; PGE_2 _= prostaglandin E_2_; RA = rheumatoid arthritis; TNFα = tumour necrosis factor α.

## Competing interests

The authors declare that they have no competing interests.

## Authors' contributions

JEJ designed the study, performed the experimental work, analysed the data and drafted the manuscript. RMA conceived of the study, participated in its design and coordination and revised the manuscript. All authors read and approved the final manuscript.
